# The nonstructural proteins of Pneumoviruses are remarkably distinct in substrate diversity and specificity

**DOI:** 10.1186/s12985-017-0881-7

**Published:** 2017-11-06

**Authors:** Michael Ribaudo, Sailen Barik

**Affiliations:** 1 Department of Biological, Geological and Environmental Sciences, and Center for Gene Regulation in Health and Disease, Cleveland State University, 2121 Euclid Avenue, Cleveland, OH 44115 USA; 2 3780 Pelham Drive Mobile, Montgomery, AL 36619 USA

**Keywords:** Paramyxovirus, Pneumovirus, Respiratory syncytial virus, Interferon, ISG

## Abstract

**Background:**

Interferon (IFN) inhibits viruses by inducing several hundred cellular genes, aptly named ‘interferon (IFN)-stimulated genes’ (ISGs). The only two RNA viruses of the *Pneumovirus* genus of the *Paramyxoviridae* family, namely Respiratory Syncytial Virus (RSV) and Pneumonia Virus of Mice (PVM), each encode two nonstructural (NS) proteins that share no sequence similarity but yet suppress IFN. Since suppression of IFN underlies the ability of these viruses to replicate in the host cells, the mechanism of such suppression has become an important area of research. This Short Report is an important extension of our previous efforts in defining this mechanism.

**Results:**

We show that, like their PVM counterparts, the RSV NS proteins also target multiple members of the ISG family. While significantly extending the substrate repertoire of the RSV NS proteins, these results, unexpectedly, also reveal that the target preferences of the NS proteins of the two viruses are entirely different. This is surprising since the two Pneumoviruses are phylogenetically close with similar genome organization and gene function, and the NS proteins of both also serve as suppressors of host IFN response.

**Conclusion:**

The finding that the NS proteins of the two highly similar viruses suppress entirely different members of the ISG family raises intriguing questions of pneumoviral NS evolution and mechanism of action.

## Introduction

IFN is recognized as an antiviral cytokine that uses STAT-family transcription factors to induce a few hundred IFN-stimulated genes (ISGs), many of which have been shown to be antiviral [1, 2]. Several RNA viruses of the *Paramyxoviridae* family, however, exhibit IFN resistance [3, 4]. We have been following this property in selected members of this family [5–9], focusing on the *Pneumovirus* genus, which is comprised of two members that are severe respiratory pathogens, namely RSV, a human virus, and PVM, a mouse virus [10, 11]. A natural mouse pathogen, PVM is lethal in laboratory mice, producing symptoms that are very similar to those of RSV disease in susceptible human subjects, whereas RSV itself replicates relatively poorly in mice, without significant mortality [10–12]. Thus, PVM infection in mice has often been recommended as an alternative model for human RSV infection [12]. Regarding innate immunity of the host, both viruses code for two nonstructural proteins, NS1 and NS2, that suppress IFN, which is essential for robust growth of the virus and the resultant pathology [13–23]. However, despite intensive research, the exact molecular mechanism of this important function of NS proteins remains unknown [6–8, 24–27]. Intriguingly, the primary structures of the NS proteins show no similarity with any other protein in biology; thus, bioinformatic analysis of their sequences offers no clue to their evolutionary origin or functional domains [6, 9, 24]. Except for the recently solved crystal structure of RSV NS1 [28], the higher order structure of the pneumoviral NS proteins has also remained unknown, in part due to difficulties of expression of the recombinant proteins; moreover, the crystal structure of RSV NS1 [28] did not offer any mechanistic insight into its IFN-suppressive function. Nonetheless, we have recently shown that the PVM NS proteins specifically degrade several mouse ISGs, namely IFITM1, TRAFD1 and ISG20 [24]. At this point, we argued that a comparison between the NS proteins of the two pneumoviruses may shed light on their structure and function. Thus, we proceeded to test if the RSV NS proteins target any ISGs, and if so, whether they are same or different from those targeted by their PVM counterparts.

## Methods

Recombinant NS clones, cell culture, transfection and immunoblot assays have been published [6, 7, 24, 29, 30] and the overall experiment followed our optimized procedure described previously [24]. In brief, codon-optimized NS1 and NS2 cDNA sequences were cloned in pCAGGS plasmid such that the proteins are expressed with FLAG tag at the amino terminus [6]. Construction of the HEK293 panel of tetracycline (Tet)-inducible FLAG-tagged ISG cells and their culture conditions have been described in detail [24, 29, 30]. The cells were grown in monolayer in Dulbecco’s minimum essential media (D-MEM), supplemented with L-glutamine, tetracycline-free fetal bovine serum (Omega Scientific, Tarzana, CA; FB-15/100; 10%), penicillin (100 IU/ml), streptomycin (100 μg/ml), hygromycin (250 μg/ml) and blasticidin (5 μg/ml). At near confluency, the cells were transfected with 1.6 μg FLAG-NS1 or 0.8 μg FLAG-NS2 plasmid or both (0.8 μg/0.8 μg), using Lipofectamine 2000 (Invitrogen/Life Technologies) following the manufacturer’s protocol. The FLAG-ISG proteins were induced with Tet (1 μg/ml) 24 h after transfection, and after another 24 h, cells were harvested for immunoblot analysis. Total cell extracts were made, and the indicated FLAG-ISG and FLAG-NS proteins were detected using FLAG antibody (Sigma, SLBF6631/F1804) as primary antibody. GAPDH served as the loading control, detected by primary mouse antibody (Santa Cruz, sc-365,062). The HRP-conjugated secondary antibody was anti-mouse, raised in goat (Santa Cruz, sc-2031). HRP was developed by ECL reaction using Prime Western Blotting Detection Reagent (GE Healthcare) and detected in the LI-COR Odyssey Fc imaging system. The band intensities in the immunoblot images were quantified and analyzed by NIH ImageJ software, and graphs were drawn by Excel using average values and standard deviations. Multiple sequence alignment and the resultant cladogram were obtained by using Clustal Omega [31].

## Results

**Fig. 1:**
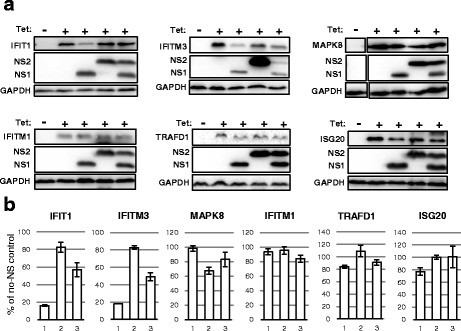
Reduction of the steady-state levels of specific ISGs by RSV NS proteins. These experiments are detailed in Methods, and representative results are shown. Briefly, Tet-inducible, stably transfected FLAG-tagged ISG cells were transiently transfected with the indicated FLAG-NS1 and -NS2 plasmids, as shown. The cells were induced with Tet (Tet +), while control cultures remained uninduced (Tet -). Immunoblotting was performed to quantify the FLAG-NS proteins and residual FLAG-ISG proteins. The immunoblot (panel **a**), and a bar graph of the ISG band intensities on the blot, expressed as percent of the ‘no-NS’ amount (panel **b**), are shown. GAPDH served as internal control, to which the ISG intensities were normalized for the plot. In panel **b**, for all ISGs, bar 1 means NS1 only, bar 2 means NS2 only, bar 3 means both NS1 and NS2 were transfected. Each bar is average of three measurements with the standard deviation shown

To start with, we examined the effect of RSV NS proteins on six major human ISGs, and the immunoblot results are shown (Fig. 1). As described previously, the original tet-inducible ISG cell panel contained 25 different ISGs that are abundantly induced by IFN [29, 30]; from these, we chose to test six because of the better growth of the cells and robust Tet-inducibility. Out of the six ISGs tested, the RSV NS proteins reduced the steady-state levels of three, namely IFIT1, IFITM3, and MAPK8 (Fig. 1), although NS1 and NS2 showed preferences, as clearly seen in Table 1.

**Table 1:** Contrasting activity of RSV and PVM nonstructural proteins on select ISGs

ISG name	GenBank#	% Reduction by RSV NS1 or NS2	% Reduction by PVM NS1 or NS2 [24]
IFIT1	NM_001548	84% NS1, 17% NS2	Not done
IFITM3	NM_021034	82% NS1, 17% NS2	Not done
MAPK8	AB451231.1	2% NS1, 33% NS2	Not done
IFITM1	NM_003641	7% NS1, 5% NS2	63% NS1, 32% NS2
TRAFD1	NM_006700	16% NS1, 0% NS2	68% NS1, 76% NS2
ISG20	NM_002201	23% NS1, 0% NS2	18% NS1, 54% NS2

This is a numerical summary of reduction of the selected ISGs by the two pneumoviral NS proteins, expressed individually. The RSV data were calculated with the average band intensities of the immunoblot of Fig. 1a, which were also graphically plotted in Fig. 1b, and the PVM data were calculated by densitometry of the corresponding immunoblot in our recent publication [24]. “Not done” indicates ISGs for which recombinant mouse homologs were unavailable. Note that in this Table we present the percent *reduction* of the ISG, whereas in Fig. 1b (bar graph), the percent *remaining* is plotted; thus 84% reduction of ISG here corresponds to 16% remaining ISG in Fig. 1b. RSV NS proteins affected the upper three ISGs are more strongly than the lower three

Whereas IFIT1 and IFITM3 were preferentially targeted by RSV NS1, MAPK8 was a preferred target of NS2. The degree of reduction was also different such that NS1 lowered the steady-state levels of IFIT1 and IFITM3 by greater than 80%, but NS2 reduced MAPK8 levels only by a third. To our surprise, however, they had little or no effect on the human counterparts of the PVM NS substrates, i.e. IFITM1, TRAFD1 and ISG20. Whereas TRAFD1 and ISG20 showed a slight sensitivity to RSV NS1 (16% and 23% reduction, respectively), they were essentially fully refractory to RSV NS2. It is to note that the PVM NS proteins also preferred as well as shared their targets to various degrees. Regardless, it seems fair to assume that screening of the full set of >400 ISGs [2] may lead to the identification of many more differential NS targets of PVM and RSV. We conclude that the full range of ISGs targeted by the pneumoviral NS proteins may be much broader than previously appreciated.

**Fig. 2:**
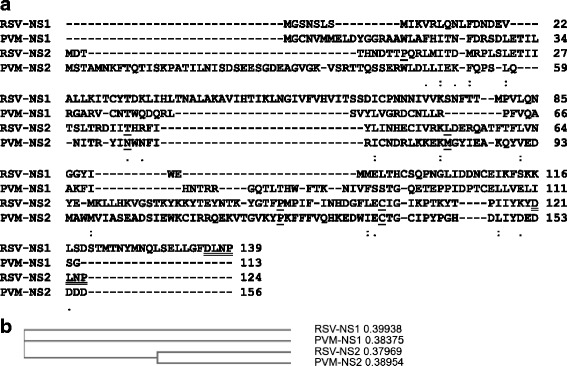
Multiple sequence alignment of the RSV and PVM NS proteins. The GenBank accession numbers are: NP_044589.1 (RSV NS1), AHW80505.1 (RSV NS2), YP_173324.1 (PVM NS1), YP_173325.1 (PVM NS2). **a** Alignment by Clustal Omega, essentially showing the lack of any significant similarity among these proteins, perhaps in agreement with their differential effect on various ISGs. Only a few conservative replacements are denoted by dots. Amino acid residues, mapped to be important for RSV NS2 ubiquitination function [32], are underlined; note that they are also conserved in PVM NS2, in spite of the high dissimilarity in the overall sequences. The C-terminal tetrapeptide DLNP, double-underlined in the two NS proteins of RSV, may have a functional role in substrate degradation, as shown before [7]; it is not found in the PVM NS sequences. **b** Standard cladogram with similarity scores, generated by the default parameters of the same program, reveals that the NS1 and NS2 orthologs of the two viruses are slightly more similar to each other than NS1 and NS2 of the same virus, perhaps suggesting that NS1 and NS2 evolved independently

Given the differential substrate choice of the NS proteins of RSV and PVM, we wondered whether NS protein sequences had any common motif or domain that might shed light on their common function as IFN antagonists. To this end, we compared the conceptually translated amino acid sequences of the NS1 and NS2 proteins of RSV and PVM, in order to find even short regions of homology. However, as shown (Fig. 2), no major sequence similarity could be discerned among all four of them. This dissimilarity of NS proteins contrasts the significant sequence similarity between the other proteins of the two viruses, which are structural proteins, such as the N (nucleocapsid) proteins sharing 61% amino acid identity, the P (phosphoprotein) proteins sharing 33% identity, and the M (matrix) proteins sharing 43% identity [11]. Nevertheless, the NS2 proteins of the two viruses deserve special mention. Previous studies from our group and others [5–7, 26] showed that NS2 of human RSV can degrade the host STAT2 protein, likely by recruiting the ubiquitin-mediated proteasome system. Very recently, extensive deletion and site-directed alanine mutagenesis of RSV NS2 protein led to the interesting finding that multiple amino acids spanning the entire length of NS2 are required for optimal ubiquitination of host proteins [32]. Five residues were identified as playing the most important role in this function (Fig. 2); in RSV NS2 sequence, they were T36, L52, P92, and C105, among which T36 was the strongest contributor [32]. As noted earlier, these residues are also conserved in the PVM NS2 sequence in the alignment (Fig. 2), the significance of which is currently unknown, since the ubiquitination function of PVM NS2 has not been investigated in depth [24, 32]. Taken together, these observations underscore diversity as well as specificity of target selection by the biologically unique pneumoviral nonstructural proteins.

## Discussion

The distinctive substrate repertoire of the two pneumoviral NS proteins agrees with their widely different primary structures, indicating that the NS proteins may have different mechanisms of action that need to be unraveled. As mentioned, there is very limited conservation of key residues, such as T36, in the NS2 proteins of the two viruses, whose significance is unknown [32]. Recently determined crystal structure of RSV NS1 showed some similarity of structural fold with the RSV M protein [28]. The significance of this exclusive similarity at the higher structural level is also enigmatic since the M protein is a virion structural protein that links the genome RNA nucleocapsid to the viral envelope, with no known role in IFN suppression. RSV NS1 promotes ubiquitin-dependent proteasomal degradation of human STAT2 [27], and in PVM, both NS1 and NS2 also have similar degradative effect on mouse STAT2 [24]. However, the lack of any sequence similarity among these proteins currently does not allow any insight into their mechanism. This is in contrast, for example, the IFN-antagonist V proteins of the Rubulavirus genus of this family, all of which degrade STAT proteins by assembling cellular proteasomes and possess conserved sequences such as a Cys-rich zinc-binding domain [33]. Although there is experimental evidence that RSV NS proteins recruit ubiquitin-dependent proteasomal complexes, the exact composition and substrate specificity of these complexes remain unknown; it is also unknown whether PVM NS proteins form similar complexes [6–9, 24, 27]. It is unlikely that there will be a common sequence motif among all the ISG substrates that the NS proteins recognize. At the same time, the NS proteins do not affect the vast majority of cellular proteins including the NS-resistant ISGs. Of note, both NS1 and NS2 of RSV have been shown to possess several other functions that need to be reconciled with their ISG-targeting activity; such functions include inhibition of apoptosis by both proteins [34, 35], regulation of cell cycle by NS1, NS2-mediated direct inhibition of RIG-I (retinoic acid-inducible gene I) [36], which is the proximal sensor of viral RNA in the IFN induction cascade, and NS1-mediated reduction of the transcriptional activity of IRF3 (IFN regulatory factor 3), which is a transcription factor for the IFN genes [37]. Clearly, the molecular mechanism of how the sequence diversity of the pneumoviral NS proteins as well as their multiple substrates still allow target specificity will be a fascinating area of future research.

At this time, we have not screened the ISGs for their ability to inhibit RSV; however, all three NS substrates reported here have a strong likelihood to function as antiviral. The IFIT (interferon-induced proteins with tetratricopeptide repeats) family in human consists of four TPR-domain proteins, of which IFIT1 is the most abundantly induced, following exposure of the cells to IFN [38]. Recent studies have revealed strong antiviral activity of IFIT1 against several viruses [38–46], including two viruses of the *Paramyxoviridae* family, i.e. PIV3 and PIV5 [29, 41]. IFIT1 inhibits the translation of mRNA with unmethylated 5′-cap [42–46] and RSV mRNA caps may be only partially methylated [47, 48]. Thus, RSV may be significantly sensitive to IFIT1-mediated translational inhibition. As proposed previously, IFIT1 may also recognize 5′-triphosphate (5′-ppp) viral RNA [49], such as the RSV leader RNA or leader-readthrough RNA [50, 51], but the outcome of these interactions cannot be easily predicted.

IFITM3, a member of the IFITM (IFN-induced transmembrane protein) family of proteins, is located in the endocytic membrane; it has been shown to strongly inhibit West Nile virus (WNV) and dengue virus (DENV) [52], but had no effect on PIV3 [29], likely because fusion of paramyxoviruses including PIV3 occurs at the cell surface without an endocytic requirement. RSV, however, is an exception in this family, as it depends on endocytic entry for the activation of its fusion protein (F) by a second proteolytic cleavage [53]. It is thus conceivable that endocytic IFITM3 may act as a roadblock to RSV entry and processing.

In contrast, IFITM1 is the only member of the IFITM family that is localized in the cell membrane [54, 55]; it is not a target of RSV NS proteins (Fig. 1) and may also have no role in RSV entry or egress. Lastly, the role of MAPK8 (Mitogen Activated Protein Kinase 8) in viral regulation may take many forms, since MAP kinases in general act as an integration point for myriad cellular signals [56], some of which may affect RSV. We do note that the ability of NS to target a host innate immune protein may not necessarily correlate with the ability of the protein to inhibit the virus, even though it makes evolutionary sense from the viral standpoint; in other words, we cannot conceive of a simple protein-protein interaction mechanism by which the NS proteins will exclusively recognize those proteins of the host cell that possess antiviral activity. Regardless, targeting the ISGs may have endowed pneumoviruses with superior IFN resistance; for example, one of the earliest comparative studies revealed that IFN concentrations of 10,000 U/ml inhibits RSV growth in cell culture by only ~15-fold while reducing PIV3 growth by 1000- to 10,000-fold [57].

## Conclusion

Our results have shown that the IFN-antagonistic pneumoviral nonstructural (NS) proteins, which are essential for optimal virus growth, suppress a much larger repertoire of host IFN-pathway factors than was previously conceived. Acting singly and/or together, the NS1 and NS2 proteins of both pneumoviruses, namely RSV and PVM, reduce the levels of multiple ISG proteins, the ultimate antiviral factors of the IFN system. The highly dissimilar and unique primary structures of the NS proteins and their ability to target equally diverse ISGs, but in a specific manner, have raised intriguing questions about their evolution and mechanisms of action.

### Limitations

We have used ISG over-expressing cell lines here, which may not produce exactly the same amount of ISG proteins as in an IFN-stimulated cell. These cell lines are also in HEK293 background, which are not physiological hosts of RSV infection. However, the RSV NS proteins can suppress a wide range of substrate concentration, expressed from diverse vectors [6, 7, 27], and can also antagonize IFN concentrations varying over a range of 500–10,000 U/ml [57]. Regarding the recombinant expression of NS proteins, we have used single amounts of the pCAGGS-NS plasmids that were pre-optimized to produce the same amounts of NS as in RSV-infected cells [6]. We used HEK293 cells, which are deficient in the STING-cGAS pathway [58, 59], to avoid spurious induction of IFN response by the transfected NS plasmids, since recent studies have discovered that this pathway is activated by cytoplasmic DNA. Finally, we have assumed that the RSV NS proteins promote degradation of the ISGs tested here, based on the proteasomal degradation of several other IFN-pathway proteins by these NS proteins, observed by several groups, as described earlier. However, our attempt to directly test this by the use of proteasomal inhibitors, such as MG132, met with technical problems because it was difficult to find an optimal concentration of MG132 that would significantly restore ISG levels without exerting cytotoxic effect on the HEK293 strain in which the ISG cell lines are created. Evidently, the mechanism of reduction of diverse ISGs by NS proteins will await further studies.

## Abbreviations

HRP: Horseradish peroxidase
IFIT: Interferon-induced proteins with tetratricopeptide repeats
IFITM: Interferon-induced transmembrane protein
IFN: Interferon
ISG: Interferon-stimulated gene
MAP K8: Mitogen-activated protein kinase 8
NS: Nonstructural
PIV3: Parainfluenza virus type 3
PVM: Pneumonia virus of mice
RSV: Respiratory syncytial virus
STAT: Signal transducers and activators of transcription
Tet: Tetracycline
TPR: Tetratricopeptide repeat
TRAFD1: TRAF-type zinc finger domain-containing protein 1
